# The impact of catheter ablation for atrial fibrillation in heart failure

**DOI:** 10.1002/joa3.12115

**Published:** 2018-09-03

**Authors:** Konstantinos Moschonas, Adam Nabeebaccus, Darlington O. Okonko, Theresa A. McDonagh, Francis D. Murgatroyd, Para Dhillon, Paul A. Scott

**Affiliations:** ^1^ Department of Cardiology King's College Hospital NHS Foundation Trust London UK; ^2^ King's College London London UK

**Keywords:** atrial fibrillation, catheter ablation, heart failure, meta‐analysis, pulmonary vein isolation

## Abstract

Atrial fibrillation (AF) commonly co‐exists with systolic heart failure (SHF) and its presence is associated with a worse prognosis. Despite this, a rhythm control approach using antiarrhythmic drugs (AADs) to reduce AF burden has demonstrated no prognostic benefit. Catheter ablation (AFA) is more effective than AADs at reducing AF burden. We performed a meta‐analysis to evaluate the impact of AFA on outcomes in SHF. Electronic databases were systematically searched. We included only randomized controlled trials that examined the impact of AFA on clinical outcomes in patients with SHF (LVEF <50%). We included studies with any ablation strategy that incorporated pulmonary vein isolation and any control group. Seven studies (n = 858) were included with a mean follow‐up of 6‐38 months. In comparison to controls, AFA was associated with significant reductions in all‐cause mortality (relative risk [RR] 0.52, *P* = 0.0009) and unplanned or heart failure hospitalization (RR 0.58, *P* < 0.00001). Compared to controls, AFA was also associated with significant improvements in LVEF (mean difference 6.30%, *P* < 0.00001), Minnesota Living with Heart Failure Questionnaire score (mean difference 9.58, *P* = 0.0003), 6‐minute walk distance (mean difference 31.78 m, *P* = 0.003) and VO_2_ max (mean difference 3.17, *P* = 0.003). However, major procedure‐related complications occurred in 2.4%‐15% of ablation patients. In patients with AF and SHF, catheter ablation has significant benefits. Further work is needed to establish the role of ablation in the routine treatment of SHF patients with AF.

## Abstract 2

We performed a meta‐analysis evaluating the impact of catheter ablation in patients with atrial fibrillation and systolic heart failure. A total of 858 patients were included across seven studies. Compared to controls, ablation was associated with lower all‐cause mortality and hospitalizations, higher LVEF, and improved symptoms and physiologic parameters.

## 1 INTRODUCTION

The incidence of heart failure continues to rise. Unfortunately, despite progress in medical and device therapy heart failure has a detrimental effect on quality of life and life expectancy.^1^ Atrial fibrillation (AF) commonly co‐exists with heart failure and its presence is associated with a worse prognosis, including increased rates of hospitalization, stroke and mortality, as well as less benefit from beta‐blockade.^2^, ^3^ However, a rhythm control approach, using antiarrhythmic drugs (AADs) to reduce the burden of AF, has not demonstrated a prognostic benefit over rate control in heart failure.^4^

Catheter ablation for AF (AFA) is more effective than AADs at reducing AF burden.^5^ The benefit of AFA in patients with heart failure was initially evaluated in four small randomized controlled trials (RCTs).^6^, ^7^, ^8^, ^9^ A meta‐analysis of these trials published in 2015, including data on 224 patients, demonstrated a significant benefit from ablation on functional and quality of life end‐points.^10^ Since the publication of this analysis, three more RCTs have been completed, using harder clinical end‐points and enrolling a further 634 patients.^11^, ^12^, ^13^ We performed this updated meta‐analysis to evaluate the impact of the additional data and specifically the effect of ablation on heart failure hospitalization and mortality.

## 2 METHODS

### 2.1 Search strategies

The electronic databases PUBMED and EMBASE were searched (until March 2018) to find primary references and reviews, together with published bibliographies and the Cochrane library. The following search terms were used: “atrial fibrillation” and (“catheter ablation” or “pulmonary vein isolation”) and (“heart failure” or “left ventricular dysfunction” or “impaired left ventricular function” or “low ejection fraction” or “cardiac failure” or “congestive heart failure”).

### 2.2 Study selection and outcomes

We selected studies that examined the impact of AFA on clinical outcomes in patients with systolic heart failure (SHF). We included only RCTs that enrolled patients with symptomatic SHF (left ventricular ejection fraction [LVEF] <50%) with at least 6 months follow‐up. We selected studies that included patients with paroxysmal atrial fibrillation (PAF) or persistent atrial fibrillation (PsAF).

We included studies with any ablation strategy that incorporated pulmonary vein isolation (PVI).

We included studies where the control group was either rate control, using medical therapy or AV node ablation, rhythm control using antiarrhythmics, or a combination of the two.

The following outcomes were evaluated:

- All‐cause mortality
- Unplanned or heart failure hospitalization
- Change in LVEF
- Change in Minnesota Living with Heart Failure Questionnaire (MLHFQ) score
- Change in 6‐minute walk distance (6MWD)
- Change in VO_2_ max

Studies in the abstract form without a published manuscript were excluded. Studies or end‐points where it was not possible to extract data were also excluded.

### 2.3 Data extraction

Studies were assessed for eligibility, and demographic and clinical outcome data were extracted by two independent investigators (KM and AN). When there were differences between observers, they reviewed the papers together to reach joint conclusions.

### 2.4 Methodological quality

Quality assessment was performed using the Cochrane Collaboration's risk of bias tool.^14^

### 2.5 Statistics

Results were analyzed using Review Manager 5.3 (Copenhagen: The Nordic Cochrane Centre, The Cochrane Collaboration, 2014). Summary estimates were calculated using the random effects model based on DerSimonian and Laird's meta‐analytic statistical method.^15^ The random effects model was chosen in view of the significant methodological heterogeneity seen between studies. We calculated risk ratios (RR) for dichotomous variables and difference in means for continuous variables.

For all meta‐analyses, Cochran's χ^2^ test and the I^2^ statistic were quantified to assess for statistical heterogeneity.

Publication bias was assessed graphically by generating a funnel plot of the logarithm of effect size against the standard error for each trial.

To explore the consistency of the results and assess for sources of heterogeneity, we performed sensitivity analyses for the end‐points of changes in LVEF, MLHFQ score and 6MWD. We did not perform sensitivity analyses for the other end‐points because of the small number of studies and/or patients reaching the end‐point in each analysis. We performed sensitivity analyses using the following grouping:

- Year of publication. We performed analyses including only studies published before 2016 and only those from 2016 onwards.
- Control group. We performed analyses including only studies that used rate control in the control group and another including only studies that used pharmacological rate control.
- AF type. We performed analyses excluding studies that included PAF patients.

In all analyses, a *P* value less than 0.05 was considered significant.

## 3 RESULTS

### 3.1 Search results

**Figure 1:**
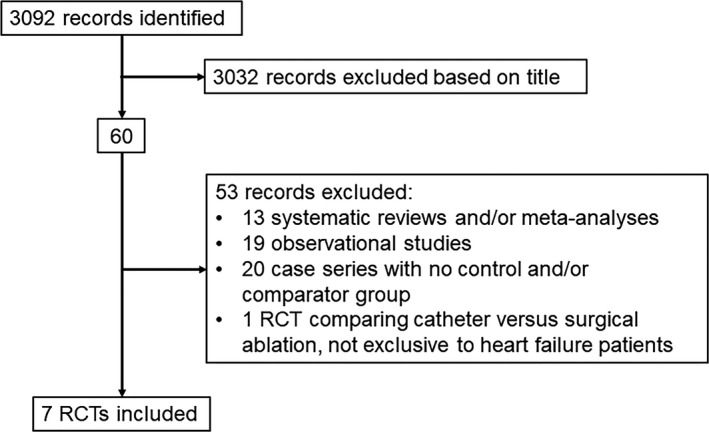
QUORUM diagram of selection process for articles included in the meta‐analysis

The search strategy yielded 3092 citations. Of these, 3032 were excluded by title/abstract and 60 retrieved for detailed evaluation. Fifty‐three further papers were excluded for the following reasons: systematic reviews or meta‐analyses (n = 13), observational studies or case‐series (n = 39), and an RCT of catheter vs surgical ablation (n = 1) (Figure 1).

Of the remaining seven studies, four reported mortality data^6^, ^7^, ^11^, ^13^, six hospitalization data^7^, ^8^, ^9^, ^11^, ^12^, ^13^, seven data on change in LVEF^6^, ^7^, ^8^, ^9^, ^11^, ^12^, ^13^, five data on change in MLWHF score^6^, ^7^, ^8^, ^9^, ^13^, six data on change in 6‐minute walk test,^7^, ^8^, ^9^, ^11^, ^12^, ^13^ and two data on change in VO2 max.^6^, ^7^

### 3.2 Study quality

Overall study quality was good (Table 1). There were, however, two areas where studies were at risk of bias. First, none of the studies attempted double blinding. While performing a sham ablation would have been difficult, this is a potential source of bias. Second, only two of the studies adequately described allocation concealment^8^, ^12^ and there is therefore a potential risk of selection bias.

**Table 1:** Risk of bias of individual studies

Area of bias	Study						
	Marrouche (2018)	Prabhu (2017)	Di Biase (2016)	Hunter (2014)	Jones (2013)	MacDonald (2011)	Khan (2008)
Selection bias							
Random sequence generation	L	L	L	L	L	L	L
Allocation concealment	U	L	U	U	U	L	U
Blinding							
Participants and personnel	S	S	S	S	S	S	S
Outcome assessment	L	L	U	L	L	L	U
Incomplete data	L	L	L	L	L	L	L
Reporting bias	L	L	L	L	L	L	L

The risk of bias was assessed using the Cochrane Collaboration's bias assessment tool.^14^

L, low risk; S, serious risk; U, unclear risk.

### 3.3 Study characteristics

The seven studies were published between 2008 and 2018. They included data on 858 patients. The mean follow‐up was 6‐38 months. Use of guideline‐directed SHF medication was high with beta‐blocker use in 76%‐97% of patients and ACE‐I/ARB in 85%‐100%. Though data were variably presented, in each of the seven studies the burden of AF was significantly lower in the AFA than control groups.

There was significant methodological heterogeneity in terms of patient characteristics, study design, and ablation strategy (Table 2).

**Table 2:** Study characteristics of randomized controlled trials investigating the effect of catheter ablation for atrial fibrillation in patients with heart failure

Study (year)	Patient no	Age	Male (%)	AF type	Cohort	HF type	Entry LA size (mm)^a^	Entry LVEF (%)^a^	Control	Ablation strategy	Multiple procedure success rate (%)		Baseline medications (%)								Follow‐up^c^	Redo rate (%) or procedure no. per patient	Procedural complications rate per patient (%)
											Ablation group	Control group	Ablation group				Control group						
													BB	ACE‐I /ARB	Dig	Amio	BB	ACE‐I/ARB	Dig	Amio			
Khan (2008)	81	60.5	92	PAF or PsAF	NYHA Class ≥II, with LVEF ≤40%	Mixed	49 ± 5	27 ± 8	AV node ablation and CRT	PVI	88^e^	0	NA								6	19.5%	2.4
MacDonald (2011)	41	63	76	PsAF	NYHA Class ≥II, with LVEF <35%	Mixed	NA	16.1 ± 7.1	Rate control	PVI ± linear lesions + CFAEs	50	0	82	95	55	NA	95	95	47	NA	6	28.5%	15.0
Jones (2013)	52	63	87	PsAF	NYHA Class ≥ II, with LVEF ≤ 35%	Mixed	50 ± 6	22 ± 8	Rate control	PVI + linear then CFAEs	88	8	92	96	62	12	92	100	46	12	12	20%	4.2
Hunter (2014)	50	57.4	96	PsAF	NYHA Class ≥II, with LVEF <50%	Mixed	52 ± 11	32 ± 8	Rate control	PVI + CFAEs then lines	81	0	NA								6	53.9%	7.7
Di Biase (2016)	203	61	74	PsAF	NYHA Class II to III, LVEF ≤40% and ICD	Mixed	47 ± 4.2	29 ± 5	Amiodarone	PVI + post wall isolation + CFAEs and linear lesions	70	34	76	92	NA	NA	80	89	NA	NA	24	1.4	2.9
Prabhu (2017)	68	60.5	91	PsAF	NYHA Class ≥II, with LVEF ≤45%	NICM	48 ± 5.5	31.8 ± 9.4	Rate control	PVI + post wall isolation	75	0	97	94	NA	NA	97	94	NA	NA	6	NA	3.0
Marrouche (2018)	363	64^d^	86	PAF or PsAF	NYHA Class ≥II, with LVEF ≤35% and ICD	Mixed	NA	32.5 (25‐38)^b^	Rate and/or rhythm control	PVI ± additional lesions at discretion of operator	63	22	93	94	18	31	95	91	31	26	37.8	24.5	3.4

AAT, antiarrhythmic therapy; ACE‐I, angiotensin converting enzyme inhibitor; Amio, amiodarone; ARB, angiotensin receptor blocker; AV, atrioventricular; BB, beta‐blockers; CFAEs, complex fractionated atrial electrograms; CRT, cardiac resynchronization therapy; Dig, digoxin; ICD, implantable cardioverter defibrillator; LVEF, left ventricular ejection fraction; MRA: Mineralocorticoid Receptor Antagonist; NA: Not available; NICM: Non‐Ischemic cardiomyopathy; NYHA: New York Heart Association; PAF: Paroxysmal Atrial Fibrillation; PsAF: Persistent Atrial Fibrillation; PVI: Pulmonary Vein Isolation.

a In the Ablation arm of the study.

b Median and interquartile range.

Amiodarone.

Agent not specified.

c Follow‐up period for primary end‐point (where different).

d Individual median for ablation and control arms.

e On or off antiarrhythmic therapy.

All studies included patients with symptomatic heart failure and reduced LVEF. Although the LVEF inclusion criteria varied from <35% to <50%, the mean entry LVEF in each study was <35%. Six studies included SHF of any etiology while the study by Prabhu et al^12^ only included patients with a nonischemic cardiomyopathy. Five studies included only patients with PsAF while the other two included PAF in addition.^9^, ^11^ In the two largest studies, all patients had an implanted ICD.^11^, ^13^

Six studies were multicenter and one single center.^6^ There was significant variation in the control groups. Five studies used rate control as the control group—one AV node ablation with biventricular pacing^9^ and four pharmacological rate control.^6^, ^7^, ^8^, ^12^ In the studies that used pharmacological rate control, the heart rate targets were either evidence‐ or guideline‐based. Of the remaining two studies, one used amiodarone^13^ and in the other “medical therapy for AF was administered in accordance with the guidelines”.^11^

There was significant variation in the study end‐points. The five smaller studies used only hemodynamic, imaging, functional, and QOL end‐points, whereas the two larger studies also presented mortality data.^11^, ^13^

All of the studies used radiofrequency energy, but only one a contact‐force catheter.^12^ In all of the studies, PVI was the cornerstone of the ablation strategy. One study performed PVI alone^9^ whereas the remaining six studies used additional substrate‐based ablation. This involved a combination of left and right atrial linear lesions, ablation of complex fractionated electrograms, and posterior LA wall isolation. In one study, the lesion set (posterior wall isolation) was clearly defined^13^, whereas in the other five the exact approach was at the discretion of the operator.

The rate of major procedural complications ranged from 2.4%‐15% per patient (not per procedure).

For four of the studies, some data not included in the original publications were presented in a subsequent meta‐analysis, which the authors of the original studies co‐authored.^10^ This supplementary data were used in our analysis where necessary.

For the end‐points of change in LVEF and change in 6MWD, the study by Marrouche et al presented a series of measurements at different time points. For our analysis, we took the 12‐month results as the number of patients contributing to the end‐point was greater and the timing more consistent with the other studies included in our analysis.^11^

### 3.4 Data synthesis

#### 3.4.1 All‐cause mortality

**Figure 2:**
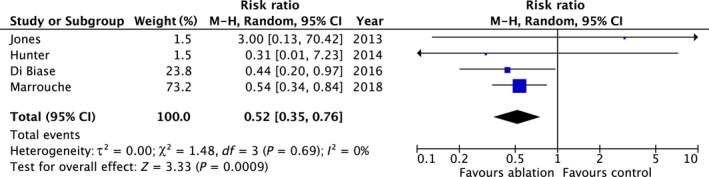
Summary of the relative risk of all‐cause mortality with AFA vs controls

Four studies (n = 668) reported all‐cause mortality data.^6^, ^7^, ^11^, ^13^ During follow‐up, there were 98 deaths. Mortality was 48% lower in AFA patients than controls (relative risk [RR] 0.52; 95% confidence intervals [CI] 0.35, 0.76; *P* = 0.0009) without statistical heterogeneity (*P* = 0.69, *I* ^2^ = 0%) (Table 3 and Figure 2).

**Table 3:** Summary estimates of relative risks and mean differences for all outcomes of AFA vs controls

Summary estimates	Relative risk (95% CI)	Patient no.	Events	No. of studies
All‐cause mortality (all studies)	0.52 (0.35, 0.76)	668	98	4
Unplanned or heart failure hospitalization (all studies)	0.58 (0.46, 0.73)	801	205	6
Summary estimates	Mean difference (95% CI)	Patient no.		No. of studies
Improvement in LVEF (%)				
All studies	6.30 (3.90, 8.71)	770		7
Newer studies	5.56 (2.15, 8.97)	551		3
Older studies	7.18 (3.39, 10.97)	219		4
Rate control as control group	8.33 (4.65, 12.02)	285		5
Pharmacological rate control as control group	8.12 (2.63, 13.61)	204		4
Only PsAF patients	6.56 (1.62, 11.51)	381		5
Improvement in MLHFQ score				
All studies	9.58 (4.45, 14.71)	396		5
Newer studies	5.00 (‐0.30, 10.30)	177		1
Older studies	11.88 (6.60, 17.15)	219		4
Rate control as control group	11.88 (6.60, 17.15)	219		4
Pharmacological rate control as control group	11.73 (3.18, 20.29)	138		3
Only PsAF patients	9.07 (2.48, 15.66)	315		4
Improvement in 6MWD				
All studies	31.78 (10.64, 52.93)	702		6
Newer studies	30.00 (‐0.40, 60.39)	537		3
Older studies	34.76 (2.87, 66.65)	165		3
Rate control only in control group	37.72 (13.11, 62.33)	231		4
Pharmacological rate control as control group	19.80 (‐12.72, 52.33)	150		3
Only PsAF patients	12.87 (2.03, 23.70)	327		4
Improvement in VO2 max score				
All studies	3.17 (1.05, 5.28)	100		2

Although the number of studies was small, the funnel plot was symmetrical.

#### 3.4.2 Unplanned or heart failure hospitalization

**Figure 3:**
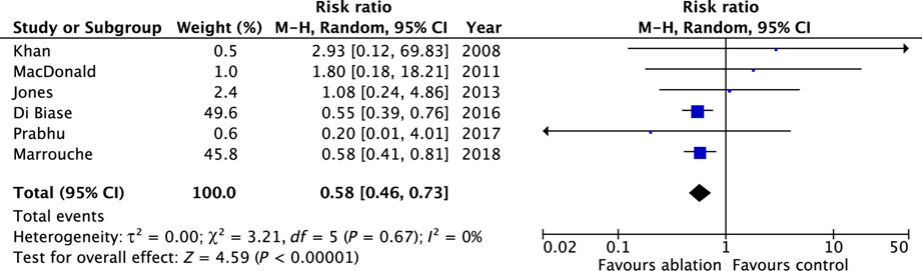
Summary of the relative risk of unplanned or heart failure hospitalization with AFA vs controls

Six studies (n = 801) reported unplanned or heart failure hospitalization.^7^, ^8^, ^9^, ^11^, ^12^, ^13^ During follow‐up, 205 patients had an unplanned or heart failure hospitalization. Hospitalization was 42% lower in AFA patients than controls (RR 0.58; 95% CI 0.46, 0.73; *P* < 0.00001) without statistical heterogeneity (*P* = 0.67, *I* ^2 ^= 0%) (Table 3 and Figure 3).

Although the number of studies was small, the funnel plot was symmetrical.

#### 3.4.3 Change in LVEF

**Figure 4:**
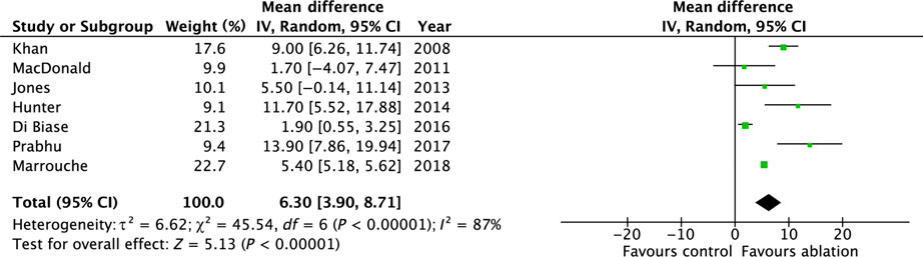
Summary of the change in LVEF with AFA vs controls

All seven studies (n = 770) reported changes in LVEF. AFA was associated with significant improvement in LVEF compared to controls (mean difference 6.30%; 95% CI 3.90, 8.71; *P* < 0.00001) with significant statistical heterogeneity (*P* < 0.00001, *I* ^2^ = 87%) (Table 3 and Figure 4).

The heterogeneity appeared sensitive to study methodology. When only studies that used rate control in the control group were included (n = 5), the result remained positive in favor of AFA (mean difference 8.33%; 95% CI 4.65, 12.02; *P* < 0.00001) with less heterogeneity (*P* = 0.03, *I* ^2^ = 62%).

The funnel plot was symmetrical.

#### 3.4.4 Change in MLHFQ Score

**Figure 5:**
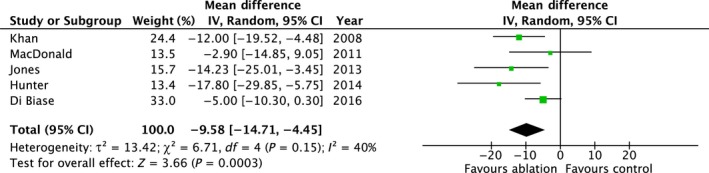
Summary of the change in MLHFQ with AFA vs controls

Five studies (n = 396) reported changes in MLHFQ score.^6^, ^7^, ^8^, ^9^, ^13^ AFA was associated with a significant improvement in MLHFQ score compared to controls (mean difference 9.58; 95% CI 4.45, 14.71; *P* = 0.0003) with moderate heterogeneity (*P* = 0.15, *I* ^2^=40%) (Table 3 and Figure 5).

The heterogeneity appeared sensitive to study methodology. Including only studies that used rate control in the control arm (n = 4) had no significant impact on the pooled result (mean difference 11.88; 95% CI 6.60, 17.15; *P* < 0.0001) but significantly reduced the statistical heterogeneity (*P* = 0.35, *I* ^2 ^= 8%).

The funnel plot was symmetrical.

#### 3.4.5 Change in 6MWD

**Figure 6:**
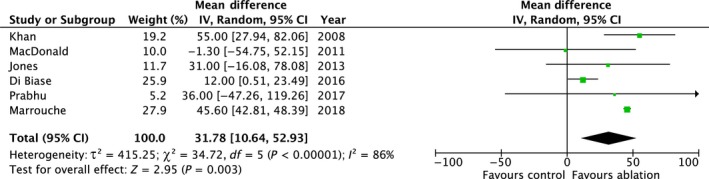
Summary of the change in 6MWD with AFA vs controls

Six studies (n = 702 patients) reported changes in 6MWD.^7^, ^8^, ^9^, ^11^, ^12^, ^13^ AFA was associated with a significant improvement in 6MWD compared to controls (mean difference 31.78 m; 95% CI 10.64, 52.93; *P* = 0.003) with significant heterogeneity (*P* < 0.00001, *I* ^2 ^= 86%) (Table 3 and Figure 6).

The heterogeneity appeared sensitive to publication date. When the three most recently published studies were excluded, the result remained positive in favor of AFA (mean difference 34.76 m; 95% CI 2.87, 66.65; *P* = 0.03) with less heterogeneity (*P* = 0.16, *I* ^2 ^= 45%).

The funnel plot was symmetrical.

#### 3.4.6 Change in VO_2_ max

**Figure 7:**
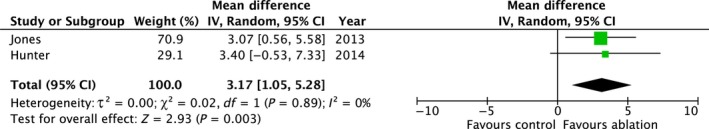
Summary of the change in VO_2_ max with AFA vs controls

Two studies (n = 100) reported on changes in VO_2_ max.^6^, ^7^ AFA was associated with a significant improvement in VO_2_ max compared to controls (mean difference 3.17; 95% CI 1.05, 5.28; *P* = 0.003) without heterogeneity (*P* = 0.89, *I* ^2 ^= 0%) (Table 3 and Figure 7).

There were too few studies to perform a meaningful funnel plot.

### 3.5 Sensitivity analyses

We performed sensitivity analyses for the end‐points of changes in LVEF, MLHFQ score, and 6MWD (Table 3). We performed analyses limited to newer studies (published from 2016 onward), older studies (published prior to 2016), studies that used only rate control in the control group, studies that used only pharmacological rate control in the control group and studies that included only patients with PsAF. For each end‐point, the results were similar to the main pooled analyses and in favor of AFA.

## 4 DISCUSSION

This meta‐analysis, including data on over 700 patients enrolled in seven RCTs, has demonstrated a significant benefit of AF ablation in patients with SHF. In patients randomized to AFA, there were significant improvements in functional capacity, quality of life, unplanned hospitalization rates, and mortality compared to controls.

Our findings are consistent with previous meta‐analyses. In 2015, Al Halabi et al combined data from four RCTs including data on 224 patients. They found that AFA was superior to rate control in improving LVEF, quality of life, and functional capacity.^10^ In 2014, Anselmino et al. published a meta‐analysis and systematic review of 26 studies, the majority of which were observational. They found significant improvements in LVEF and NT‐proBNP levels in patients treated with AFA.^16^ Our findings extend those of these previous studies, including significant more data and demonstrating a benefit of AFA on the harder end‐points of mortality and hospitalization.

AF is commonly found in patients with SHF and its prevalence increases with the severity of heart failure, affecting around 10% of patients in NYHA class I up to 50% in class IV. Furthermore, the presence of AF in patients with SHF increases the risk of hospitalization, stroke, and mortality.^2^, ^17^

Despite this, a rhythm control approach using AADs and electrical cardioversion demonstrated no significant prognostic benefit in patients with SHF, when evaluated in a large multicenter RCT. AF‐CHF randomized 1376 patients with SHF, LVEF <35% and AF, to a rhythm control approach using AADs (amiodarone, sotalol, or dofetilide), or a rate control approach.^4^ Patients in the rhythm control group were more likely to be in sinus rhythm during the study period, though 58% had at least one AF recurrence. However, there were no differences between the groups in terms of cardiovascular mortality, the primary end‐point, or any of the secondary end‐points of death, stroke, or worsening heart failure. Furthermore, when the results were reanalyzed to assess the impact of the presence of sinus rhythm, rather than being in the rhythm control group, the results were no different. The presence of sinus rhythm during follow‐up was not associated with improved outcomes compared to the presence of AF.^18^

The results of our meta‐analysis, and the studies included in it, are at odds with these findings. This is potentially explained by two factors—success rates with AFA and risks with AADs.

Although AADs have some impact on the burden of AF, AFA is significantly more effective. Khan evaluated AFA vs AADs in a meta‐analysis of 11 RCTs including 1481 patients with both paroxysmal and persistent AF. Overall AFA was associated with a 60% reduction in arrhythmia recurrence compared to AADs. There was a 48% reduction in the risk of AF in AAD‐naïve patients, and a 63% reduction in patients who had previously taken an AAD.^5^ This is supported by results of the AATAC study included in our analysis. This used amiodarone as the control arm and found that patients in the AFA arm were much more likely to have remained in sinus rhythm at the end of the study (70% vs 34%, *P* < 0.001).^19^

Antiarrhythmic drugs are well known to be associated with excess risk. In the two largest rate‐vs‐rhythm studies using AADs—AF‐CHF and AFFIRM—there was an excess of noncardiovascular mortality in the rhythm control arms.^4^, ^20^ In AFFIRM, which randomized 4060 patients to rhythm control, using AADs and cardioversion, or rate control, noncardiac mortality was significantly higher in the rhythm control arm (12% vs 8%; *P* = 0.0008).^20^ In AF‐CHF, there was also a nonsignificant increase in noncardiac mortality in the rhythm control group (8% vs 5%; *P* = 0.06).^4^ The exact mechanism of this increased mortality remains unclear, but the findings in AFFIRM were driven by increases in cancer and pulmonary deaths, while AF‐CHF found differences in rates of fatal cancer and sepsis. Furthermore, AADs are well recognized to carry a risk of ventricular pro‐arrhythmia. While the risk of a life‐threatening arrhythmia because of AAD exposure is low in patients with a structurally normal heart, the risk increases significantly in patients with reduced LVEF.^19^

The magnitude of reduction in mortality seen with ablation (49% RR reduction) in our analysis is comparable to that found in trials of ACE‐I and beta‐blockers in SHF.^21^, ^22^ Although this result is based on only two studies, enrolling 566 patients, data from the other end‐points included in our analysis support its validity. Ablation was associated with significant improvements in LVEF, 6‐minute walk and VO_2_ max, all of which are important prognostic markers in SHF. Furthermore, ablation was associated with a reduction in unplanned or heart failure hospitalization, which is again consistent with a prognostic benefit.

It is noteworthy that in all but one of the studies substrate‐based ablation in addition to PVI was performed. This included posterior wall isolation, left atrial linear lesions and ablation of complex fractionated electrograms (CFAEs). This reflects the fact that the majority of these studies were performed prior to the publication of STAR‐AF 2, which demonstrated no significant benefit of linear lesions or CFAE ablation over PVI alone in PsAF.^23^ Two of the studies presented outcome data based on whether or not patients had substrate‐based ablation in addition to PVI. In AATAC, outcomes were better in patients that underwent additional substrate‐based ablation.^13^ In contrast in CASTLE‐AF, there were no differences in outcome between the two ablation approaches.^11^ It is possible that in patients with SHF, a more aggressive ablation strategy is needed to gain maximal benefit. However, further work is needed to answer this question.

There are, however, a number of important factors that should be considered when interpreting the results of our analysis.

First, patients enrolled in the studies included in our analysis may not be representative of the population of SHF patients with AF typically encountered in clinical practice. Two‐thirds (566/851) of the patients included in our analysis came from two RCTs.^11^, ^13^ Both of these studies included only patients with symptomatic SHF and an ICD. While there are clear benefits in terms of arrhythmia monitoring of including only ICD recipients, this is a very selective patient population. Furthermore, the patients enrolled in the studies were relatively young, with mean ages ranging from 57‐64 years. In addition, it is possible that there was some degree of selection bias with investigators only enrolling patients they felt would tolerate the procedure.

Second, the procedures in these studies were performed by experienced operators in high volume centers. This is important as it cannot be assumed that lower volume operators in smaller centers would achieve the same results. Small changes in procedural success and complication rates may have a significant impact on the benefits seen with AFA in SHF.

Third, although our analysis included a significantly larger number of patients than previous reviews, the number of studies and patients is still relatively small. Furthermore, follow‐up in many studies was short with only two studies presenting follow‐up data past 12 months. This is important as SHF is a chronic problem.

Lastly, none of the studies were blinded. While performing a sham ablation would have been difficult, this is a potential source of bias and may have had some influence on the results.

It is therefore currently difficult to extrapolate these results to routine clinical practice. Further data are needed in larger more representative populations, before definitive conclusions can be drawn.

### 4.1 Limitations

Although meta‐analysis is a well‐recognized technique, it has limitations. Key among these is the difficulty in combining studies with differing methodology. The studies we included demonstrated significant methodological heterogeneity, most importantly concerning differences in ablation strategy, choice of control group and primary end‐point. Although we used a random effects model to take account of this heterogeneity and performed a number of sensitivity analyses to confirm the consistency of the results, these factors are important and need to be considered when interpreting our findings.

## 5 CONCLUSIONS

In patients with AF and SHF, catheter ablation has significant benefits over optimal medical therapy. Ablation was associated with significant improvements in functional capacity, quality of life, unplanned hospitalization rates, and mortality. Further work is needed to establish the role of ablation in the routine treatment of AF in patients with SHF.

## ACKNOWLEDGEMENTS

Nothing to report.

## CONFLICT OF INTEREST

The authors declare no conflict of interest for this article.
